# Effectiveness of Gardening-Only, Cooking-Only and Combined Cooking and Gardening Programs in Elementary Schools to Improve Fruit and Vegetable Intake: A Systematic Review

**DOI:** 10.3390/nu15133008

**Published:** 2023-06-30

**Authors:** Henna Muzaffar, Eve Guenther, Olivia Bosse, Harold Nii-Aponsah

**Affiliations:** 1 College of Health and Human Sciences, Northern Illinois University, 253 Wirtz Hall, 370 Wirtz Drive, DeKalb, IL 60115, USA; 2 Clinical Dietitian, Katherine Shaw Bethea Hospital, 1307 West Lincoln Hwy Apt 8118, Dekalb, IL 60115, USA; evemuppetshow@gmail.com; 3 The Emily Program, Eating Disorder Treatment Center, 40 Hutchinson Avenue Apt 434, Columbus, OH 43235, USA; oliviabosse18@gmail.com; 4 School of Education and Health Sciences, North Central College, 30 N Brainard Street, Naperville, IL 60540, USA; hniiaponsah@gmail.com

**Keywords:** cooking, gardening, fruits and vegetables, elementary schools, children

## Abstract

The objective is to compare the gardening, cooking, and combined cooking and gardening programs in elementary schools from the past decade (2011–2022) in improving six psychosocial and behavioral outcomes related to fruit and vegetable intake. This review was conducted following the PRISMA guidelines. Five scientific databases were searched to identify 4763 potential articles, 44 articles were retained after screening the studies’ abstract, and 36 articles were included after further investigation into each intervention. This review included 9 gardening-only programs, 8 cooking-only programs, and 19 combined cooking and gardening programs. The included studies were from 14 different countries with half of these studies took place in the United States (*n* = 18). Of the outcomes assessed, 100% (10/10) of the studies were effective in improving knowledge/skills, 90% effective in improving attitudes and self-efficacy to consume F and V (9/10), 80% produced significant results for gardening and cooking attitudes/behaviors (8/10) and willingness to try F and V (4/5), 68% (11/16) programs resulted in increase in F and V intake, and 62% (10/16) programs improved F and V preference. This review suggests that gardening-only programs (89%) and cooking-only programs (88%) were slightly more effective in producing significant findings compared to combined programs (84%), but more high-quality interventions are needed to confirm these findings.

## 1. Introduction

According to the World Health Organization (WHO), childhood obesity is considered a serious health problem worldwide. Since 1975, there has been a 10-fold increase in the prevalence of obesity (approximately 18%) in children and adolescents worldwide. Moreover, another 18% of the children are overweight [1]. In the United States (US), according to the Center of Disease Control (CDC), obesity affects one in five children, with the prevalence of obesity in children ages 6–11 being 22.2% [2]. Additionally, the 2017–2018 National Health and Nutrition Examination Survey (NHANES) in the US reported that one in six children (16.1%) between the ages of 2 and 19 were considered overweight [3]. Since this number has steadily increased globally for the past 50 years, it is important to look at the factors that contribute to this epidemic and implement creative and effective health promotion programs to combat obesity [2,4].

There are multiple causes for the high obesity prevalence in children including dietary patterns, lack of physical activity, sleep quality and quantity, illness, and genetics [2]. According to WHO and the World Cancer Research Fund, a healthy diet is characterized by an abundant intake of fruits, vegetables, and whole grains. Despite the proven health benefits of a high intake of fruits and vegetables (F and V), children and adolescents do not meet dietary recommendations for F and V [5,6]. Less than 10% of children eat the recommended number of vegetables daily and only 40% of children eat the recommended amount of fruit daily [7,8]. Low intake of F and V in children is linked to lack of access, availability, preference, and self-efficacy to consume such foods [9,10,11]. Multi-component programs that include gardening, cooking sessions, tasting sessions and educational lessons have shown to increase fruit and vegetable intake and improve F and V determinants such as preference for and willingness to taste fruits and vegetables [12].

Most children worldwide spend anywhere from 6 to 8 h of their day in school [2]. This makes schools an optimal setting for these multi-component interventions as they can be tied into the educational curriculum. Elementary school years are a logical time for healthy eating interventions with children as most eating habits are established before 15 years of age [12]. Depending on the country of residence, children may also rely on schools for 1–2 meals per day as well as snacks during the week which makes the school a prime setting for providing fruits and vegetables to the students as well as the opportunity for nutrition education, physical activity, and interactive curriculum including gardening and cooking. Provision of fruits and vegetables at home is also crucial for improving F and V intake, especially in countries where meals are not regularly provided during the school day.

Many quasi-experimental and randomized controlled trials have been conducted within the past decade to assess the effectiveness of cooking programs, gardening programs, and combined cooking and gardening programs with elementary school aged children, and furthermore, have shown mixed results. These studies have assessed a variety of outcomes related to F and V intake, including nutrition related cognitive and behavioral indicators. The hands-on school-garden based programs (SGBP) have shown potential to decrease children’s hesitancy in trying fruits and vegetables by increasing their familiarity with these foods [6]. One gardening program that targeted 2nd–5th graders in Texas included curriculum from the Junior Master Gardner (JMG) program and resulted in a significant increase in preference for fruits and vegetables as well as preference for choosing fruits and/or vegetables as a snack option [13]. Additionally, a cooking program in Colorado aimed at 4th graders resulted in significant between group improvements in fruit and vegetable preference as well as cooking self-efficacy in the intervention group compared to the control group [14]. Lastly, a combined cooking and gardening program in California showed significant improvements in self-efficacy to eat fruits and vegetables and motivation to eat fruits and vegetables but no significant results for vegetable/fruit preference or willingness to try fruits/vegetables [15]. 

To the authors’ knowledge, no other systematic review has been conducted that has focused on comparing the effectiveness of cooking, gardening, and combined cooking and gardening interventions with the elementary school population. Therefore, this systematic review has compared the available evidence from the past decade (2011–2022) for studies including gardening, cooking, and combined cooking and gardening sessions in elementary schools. This review also explored the effectiveness of key features of interventions—such as study duration, sample size, theory-based curriculum, and parental component—in achieving their desired outcomes such as fruit and vegetable intake, preference for fruits and vegetables, willingness to try fruits and vegetables, attitudes towards and self-efficacy to consume fruits and vegetables, as well as improvement in nutrition, cooking and/or gardening related knowledge, skills, attitudes and behavior.

## 2. Methods

This review included qualitative research, quantitative research, and mixed methods studies published in peer reviewed journals that evaluated the effectiveness of gardening programs and/or cooking programs with elementary/primary school aged children. The review process was conducted following the Preferred Reporting Items for Systematic Reviews and Meta-Analyses (PRISMA) guidelines [16]. An approval by an ethics committee was not needed as information was extracted from pre-existing published studies. 

### 2.1. Search Strategy

The literature search and inclusion process occurred over the span of one year from September 2021 to September 2022. Specific screening and eligibility criteria were used to determine study inclusion for this review. Five databases that were searched included the Cumulative Index to Nursing and Allied Health (CINAHL), PubMed, Web of Science, Scopus, and Academic Search Complete. Keywords used during the literature search were identical across all databases and included “cooking”, “gardening”, “programs”, “elementary schools”, and “school nutrition program”. These terms were combined with operators AND/OR and results were filtered for publication date (2011–2022), language, and population (children ages 5–12). The search results were intentionally limited to full-text academic journals and from the years 2011 to 2022 to evaluate the most recent evidence from the past decade to assess the effectiveness of cooking and gardening programs with school aged children. The reference list searches were also conducted to identify any articles missed in the database search.

After a list of articles was compiled, abstracts were screened against the inclusion criteria by three researchers (EG, HN, OB) to ensure they met the pre-determined eligibility criteria. The full text articles for the abstracts that were deemed to fit the inclusion criteria were accessed and reviewed by the same three researchers. Any discrepancies for article inclusion/exclusion were resolved by consulting with the lead author (HM). Additional details on the inclusion and exclusion criteria are presented below. 

### 2.2. Inclusion Criteria

Studies were included if they met the following inclusion criteria.

#### 2.2.1. Population

Elementary school or primary school aged children between the ages of 5 and 12 years old. Children who were outside the age range were still included if the site of the intervention was a primary or elementary school. The age range and grade classification varied depending on the school system categorization between different countries. 

#### 2.2.2. Interventions

This review consists of studies that included interventions that were primarily garden and/or cooking based. These studies had to be published between 2011 and 2022. The setting of the intervention had to be an elementary/primary school and the main target audience were children that met the age/grade requirements stated above. The definition of school setting for the purpose of this review meant that the study had to be conducted during school hours. Studies that utilized voluntary after-school activities were excluded from analysis. Student thesis papers were excluded from the literature review since they are not peer-reviewed scholarly articles. Studies were not limited to the United States; therefore, location of study was not an eligibility criterion. There were no pre-determined minimum or maximum criteria regarding the duration of selected studies.

#### 2.2.3. Outcomes

The outcomes of interest included fruit/vegetable intake, preference towards fruit and vegetables, willingness to try fruits and vegetables, attitudes towards and self-efficacy to consume fruits and vegetables, as well as improvement in nutrition, cooking and/or gardening related knowledge, skills, attitudes and behaviors. 

#### 2.2.4. Study Design 

The study designs that were included in this review were cross-sectional, observational, randomized controlled trials, randomized controlled factorial, cluster-randomized controlled trials, quasi-experimental, and mixed methods including pre/post-tests. The study design most found that met all inclusion criteria was the quasi-experimental design with pre–post evaluation. We did not limit our review based on the study designs due to the limited recent published literature on the effectiveness of cooking and gardening programs with elementary school aged children.

### 2.3. Exclusion Criteria

Studies were excluded if: (1) studies were published in a language other than English; (2) participants were children in 6th grade, or higher grades, or at the age of 13 and above; (3) study was not published between 2011 and 2022; (4) study took place outside of a school setting; (5) study did not include a cooking and/or gardening program; (6) study did not assess relevant outcomes of interest; and (7) the study was not peer reviewed.

### 2.4. Data Extraction and Synthesis

Data extraction was conducted by three researchers and any discrepancies were resolved by discussion with the lead author. The extracted data are presented in Table 1 of this review. The table is organized by program type/intervention and includes seven characteristics of each included study. These include general article information such as authors/location/year published/journal the article was published in, study design and whether a control group was utilized, sample characteristics, study intervention details, study duration, relevant outcomes, and statistical significance (*p*-value). The quantitative and qualitative evidence from the included studies were synthesized to assess which outcomes of interest were beneficially impacted by the cooking, gardening, or the combined cooking and gardening programs. 

### 2.5. Quality Appraisal of Included Studies

The quality of selected research articles was assessed by two members (EG and OB) of the research team using the GRADE criteria and any discrepancies in quality assessment ratings was resolved by the lead author (HM) [17]. The tool evaluated studies with the intention of determining the quality and certainty of evidence using seven categories which included: study design, risk of bias, inconsistency, indirectness, imprecision, publication bias, and other outcomes. Each of the questions could be scored: yes, no, cannot determine, not applicable, or not reported. Each study then received a general rating of high quality, moderate quality, low quality, or very low quality. For study design, randomized control trials (RCTs) started out as high quality and maintained this quality or were downgraded based on the remaining criteria. Any other study design started out as low quality and either was upgraded to moderate or downgraded to very low quality based on the remaining criteria. The other outcomes that may have resulted in upgrading the quality of a study included having a large effect, a strong dose response, or no plausible confounding variables. The results from the quality evaluation can be found in the last column in Table 1.

**Table 1:** Characteristics of Gardening and/or Cooking Programs in Elementary Schools.

**Article Title and Number**	**Article Information (Author, Year, Country, Journal)**	**Study Design**	**Sample**	**Study Intervention Details**	**Study Duration**	**Relevant Outcomes**	**Statistical Significance**	**Grade**
	**Gardening Programs**							
1. The Effects of Nutrition Education and Gardening on Attitudes, Preferences, and Knowledge of Minority Second to Fifth Graders in the Rio Grande Valley Towards Fruit and Vegetables	Nolan et al., 2012 [13], Rio Grande Valley of Texas, USA, HortTechnology	Quasi-experimental with pre-test and post-test; No control group	*n* = 141 (2nd–5th graders)	Junior Master Gardner (JMG) program curriculum taught by trained teachers in classrooms	7 months (August–March)	Positive change in preference for fruits/vegetables (F and V) after participation Positive change in preference for F and V as a snack choice	*p* = 0.011 *p* = 0.001	Low
2. Effects of Integrating Garden-Based Learning and E-learning into Life Education	Chen ML et al., 2013 [12], Taiwan, Life Science Journal	Quasi-experimental study; No control group	*n* = 31 (3rd grade)	40 min intervention 3 times a week, for a total of 15 weeks based on “Planting Vegetables” unit; included weekly computer class session	15 weeks	Preference for garden-based learning (GBL) and e-learning Learning effects for GBL and cooperative learning	*p* = 0.047 *p* = 0.037	Low
3. Evaluation of the Impact of A School Gardening Intervention on Children’s Fruit and Vegetable Intake: a Randomized Controlled Trial	Christian MS et al., 2014 [18], London, UK, International Journal of Behavioral Nutrition and Physical Activity	Cluster-randomized controlled trial; Control group	*n* = 641 (3–11 year old)	Royal Horticultural Society (RHS)-led vs. teacher-led (control); used curriculum based on social cognitive theory to change attitudes and behaviors related to gardening/fruits and vegetables	18 months	Borderline significant difference in combined F and V intake in unadjusted model (teacher-led had small increase) No significant difference in fruit, vegetables, or combined F and V intake between the two groups in adjusted model	*p* = 0.05 *p* = 0.06	High
4. Farm to Elementary School Programming Increases Access to Fruits and Vegetables and Increases their Consumption Among Those with Low Intake	Yoder et al., 2014 [19], Wisconsin, USA, Journal of Nutrition Education and Behavior	Quasi-experimental; No control group	*n* = 1117 (3rd–5th grade)	Used national Farm to School program/curriculum that includes logical approaches to improving students’ knowledge, attitudes, and behaviors surrounding F and V consumption	2010–2011 academic year (~10 months)	Increases in attitudes, knowledge, exposure, and willingness regarding F and V Increase in variety of F and V available on cafeteria trays	*p* < 0.001 *p* < 0.001	Low
5. School Food Gardens: Fertile Ground for Education	Beery et al., 2014 [20], Johannesburg, South Africa, Health Education	Descriptive case study; No control group	60 grade 1–7 classes (Exact participant number not reported)	School food garden project implemented by Siyakhana Initiative for Ecological Health and Food Security to promote health among young people; strong focus on health education; weekly presence/sessions	1 year	Gardens played a role in changing mindset around healthy eating and increasing knowledge of growing, eating, and preparing fruits and vegetables	*p* values not obtained d/t study design	Very low
6. Teens-As-Teachers Nutrition Program Increases Interest in Science Among Schoolchildren and Fosters Self-Efficacy in Teens	Bolshakova VLJ et al., 2018 [21], California, USA, California Agriculture	Mixed methods; control group	Intervention *n* = 71; control *n* = 22 (2nd and 3rd grade)	Healthy Living Ambassador (HLA) Program; after-school garden-based curriculum with hands-on activities to teach about nutrition, fitness, gardening and present a comprehensive and ecological approach to healthy living	10 weeks	Between groups: Increase in preference towards gardening Increase in preference towards cooking No significant increase in preference towards vegetables	*p* = 0.002 *p* = 0.044 *p* = 0.083	Moderate
7. Increasing Fruit and Vegetable Intake with Reservation and Off-Reservation Kindergarten Students in Nevada	Emm S et al., 2019 [22], Nevada, USA, Journal of Agriculture, Food Systems, and Community Development	Quasi-experimental; no control group	*n* = 45 American Indian kindergarten students *n* = 486 off-reservation kindergarten students	Veggies for Kids program which is under SNAP-Ed was taught over 12 weeks; this program uses traditional foods, tribal language, and gardening experiences to help introduce healthy eating and increase F and V intake	2017–2018 academic school year	Pre–post test difference in correctly identifying MyPlate food groups (both groups) Pre–post test difference in correctly naming selected F and V and willingness to try (off-reservation) Percentage of American Indian students who correctly named F and V and willingness to try (on-reservation)	*p* < 0.001 *p* < 0.001 *p* < 0.01	Low
8. Effect and Process Evaluation of A Real-World School Garden Program on Vegetable Consumption and its Determinants in Primary School Children	Huys N et al., 2019 [23], Ghent, Belgium, Plos One	Quasi-experimental; control group	*n* = 350 (149-intervention; 201-control); grades 1–6	‘Taste Garden’ developed by Logo Gezong+; 9-week school garden program based on intervention mapping approach and the PRECEED-PROCEED model and addresses determinants of vegetable consumption (awareness, knowledge, social influence, self-efficacy and attitudes)	~7 months (June–December)	Positive intervention effect for knowledge regarding vegetable consumption Vegetable consumption b/w groups Determinants of vegetable consumption: Attitude (not significant) Self-efficacy (significant)	*p* = 0.02 *p* < 0.01 *p* = 0.07 *p* < 0.01	Moderate
9. Effects of A School Based Intervention on Children’s Physical Activity and Healthy Eating: A Mixed-Methods Study	Khan M and Bell R, 2019 [24], London UK, International Journal of Environmental Research and Public Health	Quasi-experimental mixed methods; control group	*n* = 60 from Year 5 classes; 30 in each group	Outdoor activities related to gardening, growing of food and environmental improvement every Monday afternoon for two hours	2018–2019 academic school year	Between group differences: Daily fruit consumption Daily vegetable consumption Attitude to eating vegetables Attitude to eating fruits Preference for vegetables Preference for fruits (None were significant)	*p* = 0.728 *p* = 0.346 *p* = 0.085 *p* = 0.480 *p* = 0.078 *p* = 0.229	Moderate
	Cooking Programs							
Article Title and Number	Article Information (author, year, country, journal)	Study Design	Sample	Study Intervention Details	Study Duration	Relevant Outcomes	Statistical Significance	Grade
10. Cooking with Kids Positively Affects Fourth Graders’ Vegetable Preferences and Attitudes and Self-Efficacy for Food and Cooking	Cunningham-Sabo L and Lohse B, 2013 [14], Colorado, USA, Childhood Obesity	Randomized controlled assessment; control group	*n* = 257 (4th grade)	*Cooking with Kids* (CWK) is an experiential school-based food education program used to influence fruit/vegetable preference, food/cooking attitudes, and self-efficacy to cook; trained food educator taught cooking and tasting lessons	10 weeks (spring semester 2013)	Fruit preference (CWK vs. control) Baseline to follow-up Follow-up Vegetable preference (CWK vs. control) Baseline to follow-up Follow-up Attitudes towards food and cooking (CWK vs. control group) Baseline Baseline to follow-up Follow-up Cooking self-efficacy (CWK vs. control) Baseline to follow-up Follow-up	*p* = 0.087 *p* = 0.012 *p* = 0.007 *p* = 0.001 *p* = 0.002 *p* = 0.029 *p* < 0.001 *p* < 0.001 *p* < 0.001	High
11. Cooking Up Diversity. Impact of A Multicomponent, Multicultural, Experiential Intervention on Food and Cooking Behaviors Among Elementary-School Students from Low-Income Ethnically Diverse Families	Chen Q et al., 2014 [25], California, USA, Appetite	Mixed method quasi-experimental; control group	*n* = 604 (intervention); *n* = 600 (control) kindergarten-2nd grade	Piloted program; promoted ethnic produce through classroom food demonstrations, tastings, and home cooking activities; parents involved with home cooking activities/food kits and recipe development	February to May 2012 (4 months)	Preference for (napa cabbage, black beans, butternut squash, jicama, snap peas, bell peppers, and asparagus) Frequency of eating (napa cabbage, black beans, butternut squash, jicama, snap peas, bell peppers, and asparagus) Involvement in food preparation at home	*p* = (0.029, 0.004, <0.001, <0.001, <0.001, <0.001, and <0.001) *p* = (0.783, 0.400, 0.066, 0.008, 0.109, 0.017, and 0.001) *p* = 0.008	Low
12. Impact of A School-Based Cooking Curriculum for Fourth-Grade Students on Attitudes and Behaviors Is Influenced by Gender and Prior Cooking Experience	Cunningham-Sabo L and Lohse B, 2014 [26], USA, Journal of Nutrition Education and Behavior	Pre–post, quasi-experimental with 2 cohorts	*n* = 3135 (3rd–5th grade)	Participants were divided into 3 cohorts. One received no treatment, and the other two were exposed to either 5 2-h cooking lessons and/or 5 1-h fruit/vegetable tasting lessons.	1 year	Cooking with kids positively affected fruit and vegetable preference Independent of treatment, students without cooking experience had more than twice the gains in cooking self-efficacy Improved cooking attitudes Between groups: Total vegetable intake Total fruit intake	*p* = 0.045 and 0.033 *p* = 0.004 *p* = 0.003 *p* = 0.003 *p* = 0.490	
13. Impact of A School-Based Intervention to Promote Fruit Intake: A Cluster Randomized Controlled Trial	Rosario R et al., 2016 [27], Portugal, Public Health	Cluster randomized controlled trial; control group	*n* = 464 (6–12 years old)	Based on Health Promotion Model and social cognitive theory to encourage children to be more active and make better food selections; Twelve 3-h cooking sessions	6 months	Between groups: Fruit eaten as dessert at lunch Fruit eaten as dessert at dinner	*p* = 0.001 *p* = 0.012	High
14. Preparing and Sharing Food: A Quantitative Analysis of A Primary School-Based Food Intervention	Ensaff H et al., 2016 [28], UK, Journal of Public Health	Longitudinal comparative study; control group	*n*= 338 (3rd–6th grade)	Jamie Oliver’s Kitchen Garden Project occurred for 90 min every two weeks where they cooked and prepared dishes and were provided with recipes to take home	2012–2013 academic year	Between schools: Overall cooking experience Intervention group: Increased helping w/cooking at home Increased liking for cooking	*p* = 0.03 *p* = 0.034 *p* = 0.004	Moderate
15. Effectiveness of A Childhood Obesity Prevention Programme Delivered Through Schools, Targeting 6 and 7 Year Olds: Cluster Randomised Controlled Trial (WAVES Study)	Adab P et al., 2017 [29], United Kingdom, British Medical Journal	cluster randomized controlled trial; control group	*n* = 53 UK schools [(intervention group consisted of 26 schools and 660 students); (control group consisted of 28 schools and 732 students)]	Implementation, also known as the WAVES intervention, consisted of 2 main goals: to increase children’s physical activity and to improve their nutrient intake. Methods to reaching these goals included 30 additional minutes of physical activity, colored informative signage, cooking workshops. and a six-week cooking/sport component.	12 months	Mean BMI z-score was non-significantly lower in intervention group compared with control group No statistically significant differences between groups for fruit and vegetable intake	*p* = 0.18 *p* = 0.447	Moderate
16. Impact of A School-Based Culinary Nutrition Education Program on Vegetable Consumption Behavior, Intention, and Personal Factors Among Korean Second-Graders	Bai et al., 2018 [30], South Korea, Nutrition Research and Practice	Quasi-experimental; control group	*n* = 71 (2nd grade)	Implemented “Veggiecation” which is a nutrition education program aimed at increasing children’s acceptance and intake of vegetables through learning and engaging activities	4 weeks	Intervention group: Vegetable consumption behavior Vegetable consumption intention Vegetable consumption attitude Vegetable consumption preference Between groups: Self-efficacy to consume vegetables	*p* < 0.05 *p* < 0.05 *p* < 0.01 *p* < 0.001 *p* < 0.01	Moderate
17. Effects of A Nutrition Education Intervention on Fruit And Vegetable Consumption-Related Dietary Behavioural Factors Among Elementary School Children	Saha et al., 2020 [31], Texas, USA, Health Education Journal	Quasi-experimental; no control group	*n* = 115 (3rd–5th grade)	Curriculum based on social cognitive theory included weekly nutrition education, cooking demonstrations, and tasting sessions	6 weeks	General F and V knowledge Serving sizes of F and V Benefits of F and V consumption F and V preference F and V eating self-efficacy F and V cooking self-efficacy	All were *p* < 0.001	Low
	Cooking and Gardening Combined Programs							
Article Title and Number	Article Information (author, year, country, journal)	Study Design	Sample	Study Intervention Details	Study Duration	Relevant Outcomes	Statistical Significance	Grade
18. The Impact of a School Garden and Cooking Program on Boys’ and Girls’ Fruit and Vegetable Preferences, Taste Rating, and Intake	Jaenke et al., 2012 [32], New South Wales, Australia, Health Education and Behavior	Quasi experimental; control group	*n* = 127 (5th–6th grade)	Nutrition education curriculum, planting/tending to a garden based on social cognitive theory, production of a cookbook and participation in kitchen-based activities that included vegetables from the garden	10 weeks	Between groups Willingness to taste vegetables (boys) Fruit intake (boys/girls) Vegetables intake (boys/girls) Willingness to taste vegetables (girls)	*p* = 0.03 *p* = 0.93/<0.01 *p* = 0.67/0.72 *p* = 0.03	Moderate
19. A School Gardening and Healthy Snack Program Increased Aboriginal First Nations Children’s Preferences Towards Vegetables and Fruit	Triador et al., 2015 [33], Alberta, Canada, Journal of Nutrition Education and Behavior	Quasi-experimental; no control group	*n* = 117 (1st–6th grade)	Based on social cognitive theory; planted and tended to a garden while also preparing and eating what grew in the garden	7 months	Preference for fruits, vegetables, and fruit and vegetables combined	*p* < 0.17	Low
20. LA Sprouts: A 12-Week Gardening, Nutrition, and Cooking Randomized Control Trial Improves Determinants of Dietary Behaviors	Davis et al., 2016 [15], Los Angeles, USA, Journal of Nutrition Education and Behavior	Randomized control trial; control group	*n* = 304 (3rd–5th grade)	LA Sprouts designed and built gardens for the schools; classes taught after school for 90 min once a week including 45-min cooking/nutrition and gardening instructions lessons	12 weeks	Identification of vegetables/fruit Nutrition and gardening knowledge Increased gardening at home Vegetable/fruit preference Self-efficacy to garden Self-efficacy to cook Self-efficacy to eat fruit/vegetables Willingness to try fruit/vegetables Motivation to cook Motivation to garden Motivation to eat fruit/vegetables	*p* = 0.001/0.01 *p* = 0.003 *p* = 0.003 *p* = 0.95/0.22 *p* = 0.61 *p* = 0.71 *p* = 0.02 *p* = 0.28/0.90 *p* = 0.05 *p* = 0.04 *p* = 0.02	High
21. Impact of School-Based Vegetable Garden And Physical Activity Coordinated Health Interventions on Weight Status and Weight-Related Behaviors of Ethnically Diverse, Low-Income Students: Study Design and Baseline Data of the Texas, Grow! Eat! Go! (TGEG) Cluster-Randomized Controlled Trial	Evans et al., 2016 [34], Texas, USA, BioMed Central Public Health	Randomized control trial; control group	*n* = 1326 (3rd grade)	Coordinated Approach to Child Health (CATCH) and Learn, Grow, Eat, Go! (LGEG) program included Junior Master Gardener Health and Nutrition from the Garden and Walk Across Texas (WAT) program aspects to develop garden and physical activity curriculum based on social cognitive theory; students also participated in vegetable recipe demonstrations; 3 separate groups (WAT, LGEG, and WAT + LGEG)	5 years	LGEG Group (compared to control) Vegetable preference Vegetables exposure WAT Group (compared to control) Vegetable preference Vegetables exposure WAT + LGEG (compared to control) Vegetable preference Vegetables exposure	*p* = 0.2 *p* = 0.5 *p* = 0.2 *p* = 0.7 *p* = 0.6 *p* = 0.3	High
22. Evaluation of a Nutrition Intervention through a School-Based Food Garden to Improve Dietary Consumption, Habits and Practices in Children from the Third to Fifth Grade in Chile	Vinueza et al., 2016 [35], Santiago de Chile, Food and Nutrition Sciences	Quasi-experimental; control group	*n* = 155 (3rd–5th grade)	Used validated methodology created by “Process Mapping: Project Creation and Implementation” project in Brazil to implement garden; educational intervention included 5 workshops to familiarize with gardening, picking vegetables, nutrition education, and a final cooking workshop	6 months	Intervention Group Motivation to grow F and V at home Motivation to grow F and V in food garden Garden knowledge Taking fruit to school Purchase fruit at school Decrease in disliking cooking Control Group Motivation to grow F and V at home Motivation to grow F and V in food garden Garden knowledge Taking fruit to school Purchase fruit at school Decrease in disliking cooking	*p* = 0.1967 *p* = 0.0028 *p* < 0.0001 *p* = 0.2733 *p* < 0.0001 *p* = 0.0065 *p* = 0.0158 *p* = 0.3841 *p* = 0.0003 *p* = 0.2888 *p* = 0.6547 *p* = 0.2199	Moderate
23. LA Sprouts Randomized Controlled Nutrition, Cooking and Gardening Program Reduces Obesity and Metabolic Risk in Hispanic/Latino Youth	Gatto et al., 2017 [36], Los Angeles, USA, Pediatric Obesity	Randomized control trial; control group	*n* = 319 (3rd–5th grade)	School gardens built on campus and lessons taught for 12 weeks; each class included 45 min of interactive cooking and 45 min of a garden-based activity/lesson; based on Bandura’s “self-efficacy” construct	12 weeks	Between groups: Fruit intake Vegetable intake	*p* = 0.56 *p* = 0.04	High
24. A Multicomponent, School-Based Intervention, the Shaping Healthy Choices Program, Improves Nutrition-Related Outcomes	Scherr et al., 2017 [37], California, USA, Journal of Nutrition Education and Behavior	Randomized control; control group	*n* = 409 (4th grade)	Shaping Healthy Choices Program (SHCP) aimed at increasing nutrition knowledge and fruit/vegetable consumption and enjoyment; used Discovering Healthy Choices and Cooking Up Healthy Choice curriculum for classroom education and cooking demonstrations	1 school year	Between groups: Fruit intake Vegetable intake	*p* = 0.72 *p* = 0.26	High
25. Sowing Seeds for Healthier Diets: Children’s Perspectives on School Gardening	Nury E et al., 2017 [38], Amsterdam, International Journal of Environmental Research and Public Health	Mixed method observational study	*n* = 45 children	Study in Amsterdam. Program consisted of 25 lessons for 90 min each. Lessons consisted of indoor lessons, gardening outdoors/harvesting, cooking with produce, and indoor lessons on winter plants.	January–December 2015	Assessed via observation, formal interviews, and conversations. Children enjoyed the school gardening program Outdoor lessons more enjoyable Harvesting was the most enjoyable activity Insufficent gardening time and long expanations were disliked	No *p* Values	Very Low
26. School Gardening Increases Knowledge of Primary School Children on Edible Plants and Preferences for Vegetables	Leuven et al., 2018 [39], Utrecht, Netherlands, Food Science and Nutrition	Quasi-experimental; control group	*n* = 215 (age 10–12)	Included one classroom lesson, 15 outdoor gardening lessons, one harvesting lesson, and one cooking lesson (each 1 h); Implemented first intervention from March–October 2015 and second intervention from March-October 2016	March 2015–October 2016 (20 months)	Intervention 1 Vegetable preference (beetroot, sugar snaps, green beans, cress, and carrots) Intervention 2 Vegetable preference (potato, onion, tomato, and carrot) No significant effect on attitude towards vegetables or gardening	*p* = (<0.05, <0.05, <0.05, <0.05, and <0.01) *p* = (<0.05, <0.05, <0.05, and <0.05)	Moderate
27. Virtual Sprouts: A Virtual Gardening Pilot Intervention Increases Self-Efficacy to Cook and Eat Fruits and Vegetables in Minority Youth	Bell et al., 2018 [40], California, USA, Games for Health Journal	Quasi-experimental; control group	*n* = 180 (3rd–5th grade)	Included a virtual game 1 h/week that included special curriculum grounded in the Self Determination Theory and Social Cognitive Theory; game included gardening and cooking activities; in-class lessons included cooking demonstrations; gardening component occurred at home	3 weeks	Between groups: Self-efficacy to eat F and V Self-efficacy to cook F and V Self-efficacy to garden Vegetable intake Fruit intake Fruit preference Vegetable preference	*p* = 0.01 *p* = 0.05 *p* = 0.36 *p* = 0.38 *p* = 0.41 *p* = 0.09 *p* = 0.87	Moderate
28. Gardening Activities at School and Their Impact on Children’s Knowledge and Attitudes to the Consumption of Garden Vegetables	Kos M and Jerman J, 2019 [41], Slovenia, Problems of Education in the 21st Century	Quasi-experimental; control group	*n* = 30 (age 6–7)	Worked in school garden by weeding, growing, and gaining knowledge on the produce; consumed produce raw or within recipes such as soups, juices, and salads; activities were based on approaches of experiential and explorative learning	8 months	No significant attitude difference b/w groups regarding consumption of vegetables Intervention group Attitude towards consumption of Rocket (leafy vegetable), leek, and swede (root vegetable)	*p* = 0.003, 0.010, and 0.006	Moderate
29. Garden-Based Integrate Intervention for Improving Children’s Eating Behavior for Vegetables	Kim S and Park S, 2020 [42], Seoul, South Korea, International Journal of Environmental Research and Public Health	Experimental design; no control group	*n* = 202 (3rd and 6th grade)	12-session integrated intervention program including gardening, nutrition education, and cooking activities based on mediator model for improving children’s eating behavior for vegetables using elements of social cognitive theory; ran by certified researchers from the Korean Horticultural Therapy Association	12 weeks	3rd grade: Gardening knowledge Vegetable preference 6th grade: Gardening knowledge Vegetable preference Overall: Gardening knowledge Vegetable preference	*p* < 0.001 *p* < 0.001 *p* < 0.001 *p* < 0.001 *p* < 0.001 *p* < 0.001	Low
30. Impact of a Gardening and Physical Activity Intervention in Title 1 Schools: The TGEG Study	Berg et al., 2020 [43], Texas, USA, Childhood Obesity	Randomized control trial; control group	*n* = 1326 (3rd grade)	LGEG and WAT interventions based on Social cognitive theory (see study 19 for more details)	6 months	LGEG group: Vegetable preference WAT group: Vegetable preference LGEG + WAT group: Vegetable preference	*p* < 0.001 *p* = 0.575 *p* = 0.001	High
31. School-Based Gardening, Cooking and Nutrition Intervention Increased Vegetable Intake but Did Not Reduce BMI: Texas Sprouts—A Cluster Randomized Controlled Trial	Davis J et al., 2021, Texas [44], USA, International Journal of Behavioral Nutrition and Physical Activity	Randomized controlled cluster trial; control group	*n* = 3135 (3rd–5th grade)	Used a social ecological-transactional model and lessons were aimed to improve nutrition, cooking, and gardening knowledge/self-efficacy/attitudes as well as children’s willingness to try and their preference for F and V; included 18 one-hour lessons	1 school year (9 months)	Between groups: Vegetable intake Fruit intake	*p* = 0.002 *p* = 0.80	High
32. Testing the Effects of Two Field-to-Fork Programs on the Nutritional Outcomes of Elementary School Students from Diverse and Lower-Income Communities	Hartson K et al., 2021 [45], USA, The Journal of School Nursing	Quasi-experimental; no control group	*n* = 264	Field-to-Fork Multi-visit Program (after school component excluded due to inclusion criteria of study); included 6 in-class lessons that involved culinary lessons and use of school gardens to learn	1 academic year	Fruit consumption Vegetable consumption Knowledge of healthy cooking using vegetables	*p* = 0.138 *p* = 0.276 *p* < 0.001	Low
33. The Effects of Horticultural Activity Program on Vegetable Preference of Elementary School Students	Kim H et al., 2021 [46], Seoul, South Korea	Quasi-experimental; no control group	*n* = 136 (3rd and 5th grade)	Conducted weekly 80-min sessions of nutrition education, gardening, or cooking for 12 weeks (usually 20 min for nutrition education, 30 min for gardening/horticultural activity, and 30 min for cooking)	5 months	Gardening knowledge Vegetable preference Dietary self-efficacy (related to vegetables)	*p* < 0.001 *p* < 0.001 *p* < 0.001	Low
34. Impact of a School-based Gardening, Cooking, Nutrition Intervention on Diet Intake and Quality: The TX Sprouts Randomized Controlled Trial	Landry M. et al., 2021 [47], Texas, USA, Nutrients	Randomized controlled trial; control group	*n* = 3135 (3rd–5th grade)	Cooking With Kids (CWK) included 5, 2 h cooking and/or 5 1 h fruit and vegetable tasting lessons throughout the school year. The study examined the effects of CWK cooking and tasting curriciulum against a tasting only curriciulum.	1 year	Between groups: Total vegetable intake Total fruit intake	*p* = 0.003 *p* = 0.490	High
35. Food Environment Intervention Improves Food Knowledge, Wellbeing and Dietary Habits in Primary School Children: Project Daire, A Randomied-Controlled, Factorial Design Cluster Trial	Brennan SF et al., 2021 [48], Ireland, International Journal of Behavioral Nutrition and Physical Activity	randomized-controlled factorial design cluster trial; control group	*n* = 903 students (ages 6–7 and 10–11) from 15 different eligible schools	Organized into 4 groups/arms: Nourish, Engage, Control, and a combined Nourish and Engage group. Nourish focused primarily on cooking with locally sourced foods and Engage focused on agriculture and nutrition science. Each group was developed for both 6 to 7 year olds and 10 to 11 year olds. The control group ultimately received the Engage intervention at the end of the study.	6 months	Total Difficulties Scores (sum of all component scores) improved in all pupils who received Nourish intervention Nourish intervention produced improvements in understanding of food labels Improvements of knowledge of vegetables in season	*p* ≤ 0.02 *p* ≤0.01 *p* = 0.04	Moderate
36. Impact of a Farm-to-School Nutrition and Gardening Intervention for Native American Families from the FRESH Study: A Randomized Wait-List Controlled Trial	Taniguchi T. et al., 2022 [49], Osage Nation Regions, USA, Nutrients	Randomized wait-list controlled trial; control group	*n* = 193 (age 3–6)	Targeted tribally owned Early Childhood Education (ECE) programs and used Food Resource Equity for Sustainable Health; adapted curriculum from Early Sprouts and Watch Me Grow to create a 15-week program involving knowledge, reading, gardening, indoor/outdoor sensory activities, and cooking activities	6 months	Willingness to try (b/w groups) Tomatoes Carrots Spinach Squash Beans Peppers	*p* = 0.01 *p* = 0.50 *p* = 0.94 *p* = 0.94 *p* = 0.049 *p* = 0.91	High

## 3. Results

**Figure 1:**
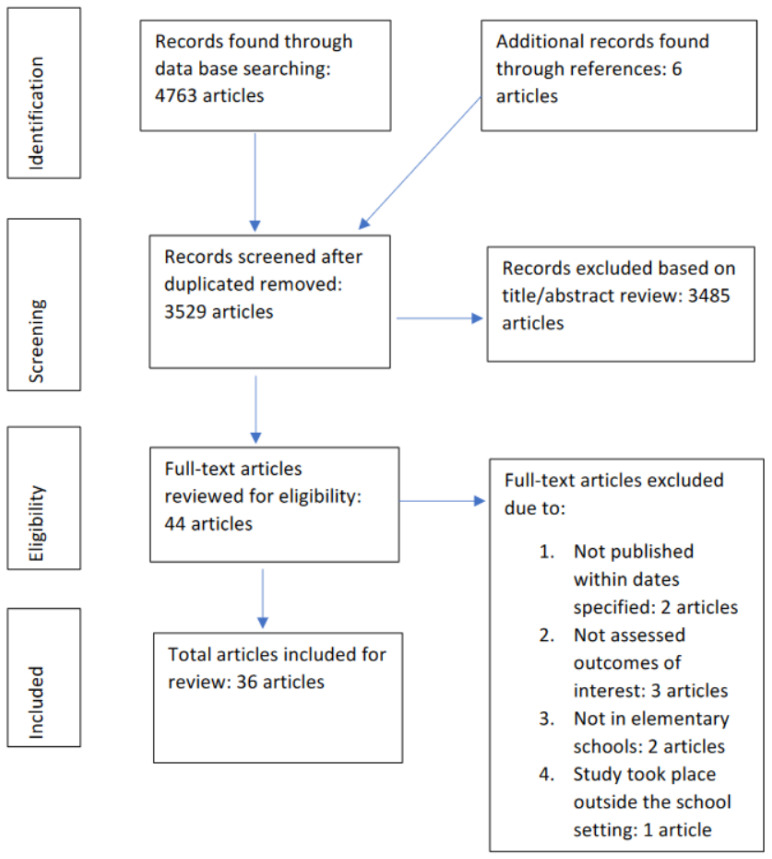
Flowchart of identification and selection of studies following the PRISMA guidelines.

CINAHL, PubMed, Web of Science, Scopus, and Academic Search Complete search engines yielded a total of 9583 articles with the keywords “gardening” or “cooking” and “elementary school”. After the initial search, search results were further narrowed by year of publication date, language, and population (elementary school children). These modifications reduced the total number of articles to 4763. Duplicate articles were eliminated, and 3529 articles were retained for further screening. Article titles and abstracts were screened for predetermined inclusion/exclusion criteria to determine eligibility and 44 potential articles were retained. After further investigation into each study’s intervention details, eight more articles were excluded. Therefore, 36 articles were included in this review as shown in Figure 1 [13,14,15,18,19,20,21,22,23,24,25,26,27,28,29,30,31,32,33,34,35,36,37,38,39,40,41,42,43,44,45,46,47,48,49,50]. The details of each intervention and article are included in Table 1. Nine of these studies included gardening-only interventions [13,18,19,20,21,22,23,24,50], eight studies included cooking-only interventions [8,19,20,21,22,23,24,25], and nineteen studies included combined cooking/gardening interventions [15,32,33,34,35,36,37,38,39,40,41,42,43,44,45,46,47,48,49]. Out of the nine gardening-only intervention studies (8/9—89% had positive results), seven studies had significant findings for at least one relevant outcome, one study reported positive qualitative findings, and one study did not have any significant results [24]. The eight cooking-only intervention studies (7/8—88% had positive results) that were included all resulted in significant findings when assessing the relevant outcomes, except one study. Out of the 19 combined cooking/gardening intervention studies (16/19—84% had positive results), 15 studies had significant findings when assessing the relevant outcomes, 1 study reported positive qualitative findings, and 3 studies did not have any significant results. For quality rating of the studies included in this review, gardening-only interventions (4/9—44% of the studies) included the least number of medium- or high-quality studies when compared with cooking-only (5/8—63% of the studies) and combined programs (14/19—74% of the studies). Overall, this review included 2 very low quality, 11 low quality, 12 moderate quality, and 11 high quality studies.

### 3.1. Study Characteristics

In total, 19,326 elementary school aged children from 36 different studies were included in this review. The included studies were from a total of 14 different countries with half of these studies taking place in the United States (*n* = 18). Seven more studies were from the United Kingdom (*n* = 4) and South Korea (*n* = 3). The remaining 11 studies were from Taiwan, South Africa, Belgium, Portugal, Canada, Chile, Netherlands, Slovenia, Australia, Ireland, and Amsterdam. The sample size of these studies ranged from 30 to 3135 participants. Studies included children from preschool to 7th grade if the intervention setting was an elementary/primary school. Therefore, participants ranged in age from 3 to 13 years old. The duration of interventions ranged from 3 weeks to 20 months. These interventions included elements such as hands-on activities, virtual nutrition education sessions, indoor/outdoor gardening activities, recipe taste-tests, take-home food kits and recipes, and cooking demonstrations. The outcome measures of these studies varied but all included the relevant outcomes of fruit/vegetable intake, preference towards fruit and vegetables, willingness to try fruits and vegetables, attitudes towards and self-efficacy to consume fruits and vegetables, as well as improvement in nutrition, cooking and/or gardening related knowledge, skills, attitudes, and behaviors. Some additional outcomes assessed in the included studies were increased variety in F and V available on cafeteria trays, correct identification of F and V, vegetable exposure, and frequency of purchasing fruit at school or bringing fruit to school. 

### 3.2. Major Findings

The impacts of gardening programs, cooking programs, and combined cooking/gardening programs on fruit/vegetable intake, preference towards fruit and vegetables, willingness to try fruits and vegetables, attitudes towards and self-efficacy to consume fruits and vegetables, as well as improvement in nutrition, cooking and/or gardening related knowledge, skills, attitudes and behaviors are all summarized in Table 1. The second to last column in this table shows the significance level (*p*-value) of the relevant outcomes from each study. A *p*-value < 0.05 was considered significant. A meta-analysis was not possible due to the variation in the study design and duration, intervention components, and outcome measurements. Table 2 includes a summary of the relevant outcomes by intervention type to better display the effectiveness of the different intervention designs. 

#### 3.2.1. Fruit and Vegetable Intake

One of the relevant outcomes of this review was fruit and vegetable (F and V) intake. Out of the nine gardening-only studies that were included, **three** reported results on this measure. One study that looked at this outcome reported no significance between group differences in daily fruit consumption (*p* = 0.728) and daily vegetable consumption (*p* = 0.346) [24]. The second study reported a significant difference in F and V intake in the teacher-led unadjusted model (*p* = 0.05), but no significant difference in fruit, vegetable, or combined F and V intake between groups in the adjusted model (*p* = 0.06) [18]. The final gardening-only study that looked at this outcome took place in Belgium and reported a significant increase in vegetable consumption between groups (*p* < 0.01) [23]. 

**Six** cooking-only intervention studies also looked at F and V intake to some extent. A mixed-methods study that took place in California reported significant increase in frequency of eating jicama, bell peppers, and asparagus (*p* = 0.008, 0.017, and 0.001) but not in frequency of eating napa cabbage, black beans, butternut squash, or snap peas (*p* = 0.783, 0.400, 0.066, and 0.109) [25]. A second study out of Portugal reported significant increases in fruit eaten as dessert at lunch (*p* = 0.001) and fruit eaten as dessert for dinner (*p* = 0.012) [27]. An additional two studies reported significant changes in vegetable consumption (*p* < 0.05) [24,25], one study indicated increase in serving sizes of F and V (*p* < 0.001) [30], and one study showed no significant increase in F and V intake (*p* = 0.447) [29]. 

**Seven** total combined cooking/gardening intervention studies included results on this outcome. One study from Australia reported a non-significant increase in fruit intake in both boys and girls (*p* = 0.67 and 0.72) and a significant increase in vegetable intake in both genders (*p* = 0.03 for both) [32]. A second study that occurred in California reported non-significance between group differences in fruit intake (*p* = 0.56) and significance between group differences in vegetable intake (*p* = 0.04) [36]. Another two combined intervention studies in the same state reported no significance between group differences in fruit intake (*p* = 0.72 and *p* = 0.41) or vegetable intake (*p* = 0.26 and *p* = 0.38) [37,40]. Another two studies in Texas also reported on this outcome and the results included significance between group differences in vegetable intake (*p* = 0.002 and *p* = 0.003) but not in fruit intake (*p* = 0.80 and 0.490) [44,47]. A final study that also took place in the USA reported on this outcome with no significant increase in intake of fruits (*p* = 0.138) or vegetables (*p* = 0.276) [45]. 

A total of 16 studies assessed this outcome, out of which 11 studies (2/3 gardening studies, 5/6 cooking studies, and 4/7 combined interventions) showed significant results. Six studies showed increase in vegetable intake, one study increased fruit intake, four studies showed increase in F and V, and five studies showed no significant findings.

#### 3.2.2. Preference for Fruits and Vegetables

Vegetable and fruit preference was another outcome of interest for this review. A total of **three** gardening-only intervention studies looked at this outcome variable. One reported a significant positive change in preference for F and V (*p* = 0.011), as well as preference for F and V as a snack choice (*p* = 0.001) [13]. The other two studies reported no significance between group differences in preference for vegetables (*p* = 0.078) or fruit (*p* = 0.229) [24] or between group increases in preference towards vegetables (*p* = 0.083) [21].

Within the cooking-only intervention studies, there were **five** studies that reported on fruit and/or vegetable preference. One study out of Colorado, reported on fruit preference between groups from baseline to follow-up (*p* = 0.087), fruit preference between groups at follow-up (*p* = 0.012), vegetable preference between groups from baseline to follow-up (*p* = 0.007), and vegetable preference between groups at follow-up (*p* = 0.001) [14]. A second study from California reported on preference for napa cabbage, black beans, butternut squash, jicama, snap peas, bell peppers, and asparagus (*p* = 0.029, 0.004, <0.001, <0.001, <0.001, <0.001, and <0.001 respectively) [25]. The final three studies reported on preference for vegetable consumption (*p* < 0.001) [30], preference for F and V (*p* < 0.001), [31] and increased fruit (*p* = 0.045) and vegetable (*p* = 0.033) preference [26]. 

A total of **eight** combined intervention studies included results on this outcome. One study from California reported no significant preference for vegetables (*p* = 0.95) or fruit (*p* = 0.22) [15]. A second study reported no significance between group differences in fruit preference (*p* = 0.09) or vegetable preference (*p* = 0.87) [40]. A third study from Canada reported no significant change in preference for fruits, vegetables, or F and V combined (*p* < 0.17) [33]. One study in Texas split the participants into three groups; however, vegetable preference was not significant in any group compared to the control group (*p* = 0.20, 0.20, and 0.60) [34]. A study in the Netherlands found significant increases in preference for beetroot, sugar snaps, green beans, cress, and carrots (*p* < 0.05, <0.05, <0.05, <0.05, and <0.01, respectively) within the first intervention along with significant increases in preference for potato, onion, tomato, and carrots (*p* < 0.05, <0.05, <0.05, and <0.05, respectively) in the second intervention [39]. A study conducted in South Korea looked at vegetable preference within 3rd grade, 6th grade, and overall (*p* < 0.001 for all variables) [42]. One study reported on vegetable preference and the results showed significant findings in two of the three intervention groups (*p* ≤ 0.001), but no significant findings in the third group (*p* = 0.575) [43]. The final study reported only on vegetable preference and the results were significant (*p* < 0.001) [46].

A total of 16 studies assessed this outcome, out of which 10 studies (1/3 gardening studies, 5/5 cooking studies, and 4/8 combined interventions) showed significant results. Six studies showed increase in preference for vegetables, four studies increased preference for F and V, and six studies showed no significant findings.

#### 3.2.3. Willingness to Try Fruits and Vegetables

The third variable of interest was willingness to try fruits and vegetables. Within the gardening-only intervention studies, a total of **two** studies reported on this outcome. The first study had significant increases in willingness regarding F and V (*p* < 0.001) [19]. The second study included students who lived off-reservation and their willingness to try F and V (*p* < 0.001) as well as students who lived on-reservation and their willingness to try F and V (*p* < 0.01) [22]. There were no cooking-only intervention studies included that assessed this outcome.

In the combined intervention study category, there were a total of **three** studies that assessed willingness to try fruit and/or vegetables. The first study looked at both willingness to try fruit and vegetables but neither result was significant (*p* = 0.28 and 0.90) [15]. A second study looked at willingness to taste vegetables by gender with the boys and girls both having significant findings (*p* = 0.03 for both) [32]. The final combined intervention study that assessed this variable looked specifically at group differences in willingness to try tomatoes, carrots, spinach, squash, beans, and peppers. The only significant results were willingness to try tomatoes and beans (*p* = 0.01 and 0.049) [49].

Five out of thirty-six studies assessed this outcome: two from the gardening-only interventions and three from the combined programs. Four out of five studies demonstrated significant results and one combined intervention did not show positive results. 

#### 3.2.4. Attitudes towards and Self-Efficacy to Consume Fruits and Vegetables

While intake of fruits and vegetables is a direct way to see the effects of cooking and/or gardening interventions on F and V consumption, the self-efficacy and attitudes towards F and V consumption are an indirect way to measure children’s psychological readiness to incorporate F and V into their diets. A total of **three** gardening-only interventions assessed attitudes and/or self-efficacy to consume F and V. One study looked at attitudes towards fruit consumption (*p* = 0.480) and attitudes towards vegetable consumption (*p* = 0.085) [24]. A second study looked at the determinants of vegetable consumption separately with attitudes towards vegetable consumption being not significant (*p* = 0.07) but self-efficacy to consume vegetables being significant (*p* < 0.01) [50]. The final study looked at a combined variable of attitudes, knowledge, exposure, and willingness to consume F and V and the results were significant (*p* < 0.001) [19].

There were also **three** cooking-only intervention studies that reported on this outcome. The first study looked at attitudes towards vegetable consumption (*p* < 0.01) and intention to consume vegetables (*p* < 0.05) within the intervention group as well as between group differences in self-efficacy to consume vegetables (*p* < 0.01) [30]. The second study only reported on self-efficacy to eat F and V and the results were also significant (*p* < 0.001) [31]. The third study looked at attitudes towards cooking and the results was significant (*p* = 0.003) [26]. 

Within the combined intervention studies, there were a total of **four** studies that assessed this outcome. One of the studies assessed self-efficacy to eat F and V (*p* = 0.02) as well as motivation to eat F and V (*p* = 0.02) [15]. Another study only looked at the self-efficacy to eat F and V and the results were also significant (*p* = 0.01) [40]. The third study also found a significant result regarding dietary self-efficacy to consume vegetables (*p* < 0.001) [46]. The fourth and final study found no significant attitude difference between groups regarding consumption of vegetables but significant attitudes towards consumption of rocket, leek, and swede (*p* = 0.003, 0.010, and 0.006 respectively) in the intervention group [41].

Ten out of thirty-six studies assessed this outcome: three from the gardening-only interventions, three from cooking-only interventions and four from the combined programs. Nine out of ten studies demonstrated significant results and one gardening-only intervention did not show positive results.

#### 3.2.5. Nutrition, Cooking and/or Gardening Knowledge and Skills 

While fruit and vegetable intake and related psychosocial aspects were the main outcomes of interest, another outcome looked at increase in nutrition, cooking, and gardening knowledge and skills. There was a total of **two** gardening-only intervention studies that assessed this variable. The first study reported that gardens played a role in increasing knowledge of growing, eating, and preparing fruits and vegetables [13], while the second study showed significant learning effects for garden-based learning (GBL) and cooperative learning (*p* = 0.037) [50]. 

There was only **one** cooking-only intervention study that reported results on this variable. This study looked at the overall amount of cooking experience between schools and the results were significant (*p* = 0.03) [28].

Within the combined intervention studies, there were a total of **seven** studies that assessed this variable. The first study reported significant increases in nutrition and gardening knowledge (*p* = 0.003) [15]. A second study looked at knowledge of healthy cooking using vegetables and the results were significant (*p* < 0.001) [45]. The third article assessed gardening knowledge and looked at 3rd grade gardening knowledge (*p* < 0.001), 6th grade gardening knowledge (*p* < 0.001), and overall gardening knowledge (*p* < 0.001) [42]. The fourth study that assessed this variable reported on gardening knowledge as well; the results were also significant (*p* < 0.001) [46]. The fifth article looked at the gardening knowledge in both the intervention (*p* < 0.0001) and the control groups (*p* = 0.0003) [41]. The sixth article reported significant increase for the treatment group in improvement of knowledge of vegetables in season (*p* = 0.04) [48]. The final article reported positive qualitative evaluation for knowledge gained and activities enjoyed from the program [38]. 

Ten out of thirty-six studies assessed this outcome: two from the gardening-only interventions, one from cooking-only interventions, and seven from the combined programs. All studies demonstrated significant results for increase in nutrition, cooking, and/or gardening knowledge and skills.

#### 3.2.6. Cooking/Gardening Related Attitudes and Behaviors

The final outcome that was assessed in this review was cooking and/or gardening attitudes and behaviors. There were **three** gardening-only interventions that assessed attitudes and behaviors related to gardening. The first study was qualitative in design and reported an overall conclusion that gardens played a role in changing the mindset around healthy eating [20]. A second study showed a significant increase in preference towards both gardening and cooking [20]. The final study showed a significant preference for garden-based and e-learning (*p* = 0.047) [50].

There were **four** cooking-only intervention studies that reported on this outcome. The first article assessed attitudes towards cooking between groups at baseline (*p* = 0.002), from baseline to follow-up (*p* = 0.029), and at follow-up (*p* < 0.001), as well as cooking self-efficacy between groups from baseline to follow-up (*p* < 0.001) and at follow-up (*p* < 0.001) [14]. The second study reported significant involvement in food preparation at home (*p* = 0.008) [25]. The third study reported results on a significant increase in liking for cooking in the intervention group (*p* = 0.004), as well as increased helping with cooking at home in the intervention group (*p* = 0.034) [28]. In the final study, the cooking self-efficacy increased significantly (*p* = 0.004) [26].

Within the combined intervention studies, there were a total of **four** studies that assessed cooking and/or gardening related attitudes and behaviors. One of the studies reported significant increase in gardening at home (*p* = 0.003), motivation to cook (*p* = 0.05), and motivation to garden (*p* = 0.04) [15]. Another study reported significant between group differences in self-efficacy to cook F and V (*p* = 0.05), but not in self-efficacy to garden (*p* = 0.36) [40], while a third study showed no significant effect on attitudes towards gardening [34]. The final study assessed motivation to grow F and V at home for the intervention group (*p* = 0.1967) and the control group (*p* = 0.0158), as well as motivation to grow F and V in a food garden for the intervention group (*p* = 0.0028) and control group (*p* = 0.3841). This same study also reported a decrease in the disliking of cooking in the intervention group (*p* = 0.0065) and the control group (*p* = 0.2199) [35]. 

Eleven out of thirty-six studies assessed this outcome: three from the gardening-only interventions, four from cooking-only interventions, and four from the combined programs. Ten out of eleven studies demonstrated significant results and one combined intervention did not show positive results.

## 4. Discussion

In this review, the results from the 36 included studies indicated that gardening-only and cooking-only were somewhat more effective than multi-component programs in improving F and V intake and related cognitive and psychosocial variables; however, all three types of programs are promising and effective strategies. Over 80% of the studies in all three categories yielded significant/positive findings. The gardening-only and cooking-only interventions assessed one to three outcomes of interest and the combined interventions assessed up to five outcomes of interest. Seemingly, the gardening-only intervention had the most impact on willingness to try F and V out of the three intervention types. Cooking-only programs produced most consistent significant findings for all the outcome measures that were assessed. The combined cooking and gardening interventions showed they were consistently effective in increasing nutrition, cooking/gardening related knowledge and skills and improving attitudes and self-efficacy to consume F and V. 

Intervention effects on F and V intake indicated that cooking-only programs (5/6 programs) were the most consistently effective in improving this outcome. Cooking interventions are usually paired with tasting new foods/recipes which can explain why they may be more effective with increases in consumption of those healthy foods due to the taste exposure in the intervention [51]. Most of the gardening-only interventions (2/3 studies) also showed improvement related to this outcome. The results indicated that the multi-component combined cooking and gardening interventions (4/7 studies) were the least effective in improving F and V intake between the three types of intervention. In terms of the effectiveness of the different interventions on preference for F and V, cooking-only programs were also the most consistently effective in improving this outcome and all five programs demonstrated significant findings. Out of the three gardening-only programs that assessed this outcome, two had non-significant results. The combined interventions were moderately effective for this outcome with 50% of these interventions producing significant results. Thus, evidence from this review suggests that cooking-only interventions are most consistently effective in increasing the preference for F and V and the intake of especially vegetables. The increased effectiveness of cooking intervention for F and V intake and preference may be due the fact that 7/8 cooking studies in this review included a tasting component as compared to 4/9 gardening studies. A review by Robinson-O’Brien et al. also suggests that gardening programs can improve preference and intake of F and V if they include tasting activities [4]. According to Charlton et al., vegetable intake is one of the most difficult dietary behaviors to change and may be possible with experiential learning activities such as cooking and gardening when paired with nutrition education and tasting experiences [51].

Intervention effects on willingness to try F and V indicated that gardening-only interventions were consistently effective as both programs that assessed this outcome had significant results. There were no cooking-only programs that assessed this objective. The combined cooking/gardening interventions were also moderately effective with two out of the three studies assessing this variable producing significant findings. The effectiveness of the different types of interventions on attitudes towards and self-efficacy to consume F and V showed that the three cooking-only and four combined interventions were able to produce significant findings. For gardening-only programs, two out of the three studies resulted in significant findings. These results suggest that most studies included in this review assessing willingness to try F and V, attitudes towards F and V, and self-efficacy to consume F and V had significant positive findings. 

Another outcome that was assessed in this review was nutrition, cooking, and gardening knowledge. Knowledge gain increases a person’s potential and ability to make healthier food choices later [8]. Ten out of thirty-six studies included in this review included evaluation of change in knowledge. All three types of studies that assessed this outcome showed significant findings and suggest that experiential learning programs incorporating cooking and/or gardening can result in gain in knowledge and skills. Intervention effects on cooking/gardening related attitudes and behaviors indicated that both cooking-only and gardening-only interventions were consistently effective in changing the targeted behaviors. The combined intervention types were also effective in successfully bringing about a change in this target variable as three out of the four combined studies showed significant findings for this outcome. Knowledge, attitudes, and preferences are easier to change than changing the actual behavior. Longer interventions, more intense and frequent sessions and long-term follow up may improve the efficacy of interventions in changing behaviors [51]. 

### 4.1. Comparison of Results for Different Study Characteristics

#### 4.1.1. Length of Intervention

All 36 studies were separated into three categories to assess whether the length of the intervention had any effect on the results of the studies. These categories were formed using three ranges (1 week–6 months, 7–12 months, and greater than 1 year). There was a total of 18 interventions that lasted 1 week–6 months. Out of these 18 interventions, all the studies (100%) produced at least one significant finding [14,15,21,25,27,30,31,32,35,36,38,40,42,43,46,48,49,50]. For the next category, there were a total of 15 studies that had a duration from 7 to 12 months [13,19,20,22,23,24,26,28,29,33,37,41,44,45,47]. Out of these 15 studies, 10 (66%) produced at least one significant finding [13,19,22,23,25,26,41,44,45,47]. The final category included studies with a duration greater than one year. There was a total of three studies that fit into this category [18,34,39] and two (66%) produced at least one significant finding [18,39]. Results from this review suggest that shorter interventions lasting 6 months or less may be most effective in bringing about positive changes in F and V intake and associated variables as longer interventions may lose rigor and participant’s interest. These findings are in line with the review conducted by Chan et al., which also suggested that shorter interventions were more effective in increasing F and V intake [12]. A review by Charlton et al. also acknowledges that longer programs may not be always feasible due to time constraints and limited resources [51].

#### 4.1.2. Sample Size

The sample size variable was compared similarly by dividing the studies into three categories. These categories included 0–499 students, 500–1000 students, and greater than 1000 students. There was a total of 25 studies that had 0–499 students in participation [13,14,15,20,21,23,24,27,28,30,31,32,33,35,36,37,38,39,40,41,42,45,46,49,50]. Out of these studies, a total of 20 studies (80%) produced at least one significant result [13,14,15,21,23,27,28,30,31,32,35,36,39,40,41,42,45,46,49,50]. Within the 500–1000 participant category, there were a total of three studies. All three studies (100%) produced at least one significant result [18,22,48]. In the final category, there were a total of eight studies that had a sample size greater than 1000 students [19,25,26,29,34,43,44,47]. Out of these eight studies, six (75%) produced at least one significant result [19,25,26,43,44,47]. These results suggest that studies with sample sizes between 500 and 1000 may be more promising than the other two categories in bringing about significant changes; however, this result must be interpreted with caution as we had only three studies in this review falling in the 500–1000 sample size category.

#### 4.1.3. Theory-Based Intervention 

The third study characteristic that was assessed was comparison of theory-based interventions with non-theory based interventions. Out of the 36 total studies, 14 (39%) included theory-based interventions [18,23,27,31,32,33,34,35,36,40,42,43,44,47]. The most common theory used in the interventions was the social cognitive theory with eight studies incorporating this theory into their interventions [18,31,32,33,34,40,42,43]. Other theories that were used included the PRECEED-PROCEED model [23], the Health Promotion Model [27], the self-determination theory [40], the social ecological-transactional model (SET-M) [44,47], and process mapping [35]. Out of the 14 studies that were theory based, a total of 12 (86%) produced at least one significant result [18,23,27,31,32,35,36,40,42,43,44,47]. The remaining 22 non-theory-based studies had a total of 18 (82%) studies that produced at least one significant result [13,14,15,19,21,22,25,26,28,30,38,39,41,45,46,48,49,50]. With these results being so close, it is inconclusive whether interventions that are theory based are more effective than those that are not theory based. 

#### 4.1.4. Parental Involvement

Another study characteristic that was assessed was parental component vs. no parental component in the included studies. Only seven studies (2/9 in gardening programs, 2/8 in cooking programs, and 3/9 in the combined programs) in this review included some parental component. However, all these studies did not explain the parental component in adequate detail and 5/7 of these studies seemed to have low parental involvement. It is noteworthy that 6/7 of these interventions had significant findings related to F and V intake, preference for F and V, and willingness to try F and V. Parental involvement seems to be a promising strategy for changing food related behaviors especially for elementary school children which is in accordance with the results from the reviews conducted by Charlton et al. and Tomayko et al. [51,52]

### 4.2. Strengths and Limitations

One of the strengths of this review is the in-depth search of the literature that was performed by three researchers and any discrepancies resolved by discussion with the lead author. The search included all relevant databases which resulted in a reduction of selection bias by identifying most of the relevant literature. Additionally, the GRADE criteria were used to identify the quality of each article. However, we must acknowledge the limitations that we encountered as we collected the data and analyzed the information for summarizing the available evidence and making recommendations for future health promotion programs with children. First, not all the included studies assessed each of the six main outcomes that were of interest to this review. Also, a variety of evaluation tools were used by the studies for the same outcome. This makes it difficult to fully assess which type of intervention has the most potential to be beneficial for our selected outcomes of interest. Secondly, information about intervention components, sample size, study duration, program delivery, teachers/program staff training, and any parental involvement varied across all intervention types and studies. This heterogeneity in study designs made it impossible to conduct meta-analysis and hence we have shared a general sum up of findings in this review. Thirdly, the included 36 studies took place in 14 different countries, with the majority (24 studies) being from the United States, United Kingdom, or South Korea. Thus, caution must be exercised in generalizing the results of this review to other countries, as we had limited information from 11 other countries from where we identified only one program encompassing cooking/gardening components. This variance in location also contributed to an inconsistency in what age range was included in the criteria of elementary or primary school-aged children as some countries include children younger than 5 years old and older than 12 years old in this population. Moreover, not all countries offer meals and snacks during the school day. Regardless of meal provision, cooking and gardening programs in schools offer a promising strategy to inculcate healthy eating behaviors in young children. 

## 5. Conclusions

The findings of this systematic review indicate that cooking-only interventions and gardening-only interventions seemingly were more effective in positively targeting at least one of the six main outcomes assessed in this review when compared with combined cooking and gardening interventions, but all three types of programs are promising strategies to promote F and V intake or related psychosocial variables. The cooking-only interventions produced the most significant results for three of the outcomes (F and V intake, preference for F and V, and cooking/gardening related attitudes and behaviors) and were equally as effective as the combined intervention for an additional two objectives (attitudes towards and/or self-efficacy to consume F and V, and nutrition, cooking and gardening knowledge and skills). Gardening-only interventions have shown most consistent results with increasing willingness to try fruits and vegetables. Furthermore, sample size, length of the intervention and parental involvement were shown to have potential effects on the production of significant results. However, the interventions that were theory based were not shown to be more effective than those interventions that were not theory based. Future robust cooking and/or gardening based interventions are recommended to further assess these variables and effectively promote fruit and vegetable consumption in elementary school aged children. More published literature is needed that encompasses details of program content and assessment tools, use of any theoretical framework to guide the program, mode of delivery, training of program staff, and process evaluation of the delivered interventions. In addition, more programs are needed with consistent study designs in multiple natural settings such as schools, home, and the community, and that which include parental components as more and more of the literature suggests increased effectiveness of elementary school programs to promote healthy eating that have parental involvement across multiple sectors and environments [52]. Moreover, increasing F and V provision at school and home may also be crucial in affecting the food choices of children, as it is not possible for children to initiate and sustain healthy eating without having access to increased F and V and other healthy foods.

## Figures and Tables

**Table 2:** Relevant Outcomes by Intervention Type.

**Article**	**F and V Intake**	**Preference for F and V**	**Willingness to Try F and V**	**Attitudes Towards and/or Self-Efficacy to Consume F and V**	**Cooking/Gardening Related Behaviors**	**Cooking and/or Gardening Knowledge/Skills**
***Gardening-Only Interventions***						
1	Not assessed	Significant	Not assessed	Not assessed	Not assessed	Not assessed
2	Not assessed	Not assessed	Not assessed	Not assessed	Significant	Significant
3	Significant (unadjusted model)	Not assessed	Not assessed	Not assessed	Not assessed	Not assessed
4	Not assessed	Not assessed	Significant	Significant	Not assessed	Not assessed
5	Not assessed	Not assessed	Not assessed	Not assessed	Not assessed	Assessed (positive qualitative evaluation)
6	Not assessed	Not significant	Not assessed	Not assessed	Significant	Not assessed
7	Not assessed	Not assessed	Significant	Not assessed	Not assessed	Not assessed
8	Significant	Not assessed	Not assessed	Significant (self-efficacy) Not significant (attitudes)	Not assessed	Not assessed
9	Not significant	Not significant	Not assessed	Not significant	Not assessed	Not assessed
Total assessed	3	3	2	3	2	1
Total Significant	2	1	2	2	2	1
**Article**	**F and V Intake**	**Preference for F and V**	**Willingness to try F and V**	**Attitudes Towards and/or Self-Efficacy to Consume F and V**	**Cooking/Gardening Related Behaviors**	**Cooking and/or Gardening Knowledge/Skills**
***Cooking-Only Interventions***						
10	Not assessed	Significant	Not assessed	Not assessed	Significant	Not assessed
11	Significant (for some vegetables)	Significant	Not assessed	Not assessed	Significant	Not assessed
12	Significant	Not assessed	Not assessed	Not assessed	Not assessed	Not assessed
13	Not assessed	Not assessed	Not assessed	Not assessed	Significant	Significant
14	Significant	Significant	Not assessed	Significant	Not assessed	Not assessed
15	Significant	Significant	Not assessed	Significant	Not assessed	Significant
16	Significant	Significant	Not assessed	Significiant	Significant	Not assessed
17	Not significant	Not assessed	Not assessed	Not assessed	Not assessed	Not assessed
Total assessed	6	5	0	3	4	2
Total Significant	5	5	0	3	4	2
**Article**	**F and V Intake**	**Preference for F and V**	**Willingness to try F and V**	**Attitudes Towards and/or Self-Efficacy to Consume F and V**	**Cooking/Gardening Related Behaviors**	**Nutrition, Cooking and/or Gardening Knowledge/Skills**
***Cooking and Gardening Combined Interventions***						
18	Significant (fruit intake in girls)	Not assessed	Significant	Not assessed	Not assessed	Not assessed
19	Not assessed	Not significant	Not assessed	Not assessed	Not assessed	Not assessed
20	Not assessed	Not significant	Not significant	Significant	Significant	Significant
21	Not assessed	Not significant	Not assessed	Not assessed	Not assessed	Not assessed
22	Not assessed	Not assessed	Not assessed	Not assessed	Significant	Significant
23	Significant (vegetables only)	Not assessed	Not assessed	Not assessed	Not assessed	Not assessed
24	Not significant	Not assessed	Not assessed	Not assessed	Not assessed	Not assessed
25	Not assessed	Significant	Not assessed	Not assessed	Not significant	Not assessed
26	Not significant	Not significant	Not assessed	Significant	Not significant	Not assessed
27	Not assessed	Not assessed	Not assessed	Significant	Not assessed	Not assessed
28	Not assessed	Significant	Not assessed	Not assessed	Not assessed	Significant
29	Not assessed	Significant	Not assessed	Not assessed	Not assessed	Not assessed
30	Significant (vegetables only)	Not assessed	Not assessed	Not assessed	Not assessed	Not assessed
31	Not significant	Not assessed	Not assessed	Not assessed	Not assessed	Significant
32	Not assessed	Significant	Not assessed	Significant	Not assessed	Significant
33	Significant (vegetables only)	Not assessed	Not assessed	Not assessed	Not assessed	Not assessed
34	Not assessed	Not assessed	Significant (beans and tomatoes only)	Not assessed	Not assessed	Not assessed
35	Not assessed	Not assessed	Not assessed	Not assessed	Not assessed	Significant
36	Not assessed	Not assessed	Not assessed	Not assessed	Not assessed	Assessed (qualitative results)
Total assessed	7	8	3	4	4	6
Total Significant	4	4	2	4	2	6

## Data Availability

Authors do not have any additional data to share as the systematic review looked at studies already published and no new study was conducted.
